# Mitochondrial Fusion Protein Mfn2 and Its Role in Heart Failure

**DOI:** 10.3389/fmolb.2021.681237

**Published:** 2021-05-07

**Authors:** Lei Chen, Bilin Liu, Yuan Qin, Anqi Li, Meng Gao, Hanyu Liu, Guohua Gong

**Affiliations:** ^1^ Institute for Regenerative Medicine, Shanghai East Hospital, School of Life Sciences and Technology, Tongji University, Shanghai, China; ^2^ Department of Pharmacy, Shanghai East Hospital, Tongji University, Shanghai, China; ^3^ Department of Gastroenterology, Shanghai East Hospital, School of Life Sciences and Technology, Tongji University, Shanghai, China

**Keywords:** Mfn2, mitochondria fusion, endoplasmic reticulum–mitochondria contacts, mitophagy, heart failure

## Abstract

Mitofusin 2 (Mfn2) is a transmembrane GTPase located on the mitochondrial outer membrane that contributes to mitochondrial network regulation. It is an essential multifunctional protein that participates in various biological processes under physical and pathological conditions, including mitochondrial fusion, reticulum–mitochondria contacts, mitochondrial quality control, and apoptosis. Mfn2 dysfunctions have been found to contribute to cardiovascular diseases, such as ischemia-reperfusion injury, heart failure, and dilated cardiomyopathy. Here, this review mainly focuses on what is known about the structure and function of Mfn2 and its crucial role in heart failure.

## Introduction

In mammals, cardiomyocytes are highly metabolically active cells. Mitochondria, as a cellular energy power plant, produce ∼90% cellular ATP and occupy ∼40% of the adult cardiomyocyte volume (Marin-Garcia and Akhmedov, 2016). The dysfunctional mitochondria have been linked with various heart diseases (Ong et al., 2012; Dorn, 2013). In most cells, mitochondria are highly dynamic organelles organized in a tubular, dynamic network that constantly undergo movement, fusion, and fission events. Adult cardiomyocytes exhibit a fragmented mitochondrial network. Spheroid mitochondria are densely confined among myofibrils—limiting the movement of mitochondria (Gong et al., 2015), which leads to the low frequency of mitochondrial dynamics events, especially mitochondrial fission and fusion (Eisner et al., 2017; Qin et al., 2018).

Mitochondria fusion and fission, termed as major mitochondrial dynamics, are mediated by mitofusin1/2 (Mfn1/2), optic atrophy 1 (Opa1), and dynamin-related protein 1 (Drp1). Although mitochondrial dynamics events are rare in adult cardiomyocytes (Qin et al., 2018), these dynamism proteins are highly expressed in the adult cardiac.

Mitofusin 2 (Mfn2) is abundantly expressed in the heart, in which it is located at the outer mitochondrial membrane (OMM) to modulate its fusion (Eura et al., 2003). It has been found that Mfn2 participates in multiple cellular biological processes under physical and pathological conditions. Apart from mitochondria fusion, Mfn2 is also involved in the endoplasmic reticulum (ER)–mitochondria tether, mitophagy, cellular apoptosis, and proliferation. Mfn2 as the central player of the Charcot Marie tooth disease, also participated in the onset and development of various heart diseases, including transverse aortic constriction (TAC), induced heart failure, and myocardial infarction (MI) (Givvimani et al., 2014).

In this review, we focus on the current understanding of Mfn2 structure, function, and its role in heart failure.

## The Structure of Mitofusin 2

**FIGURE 1:**
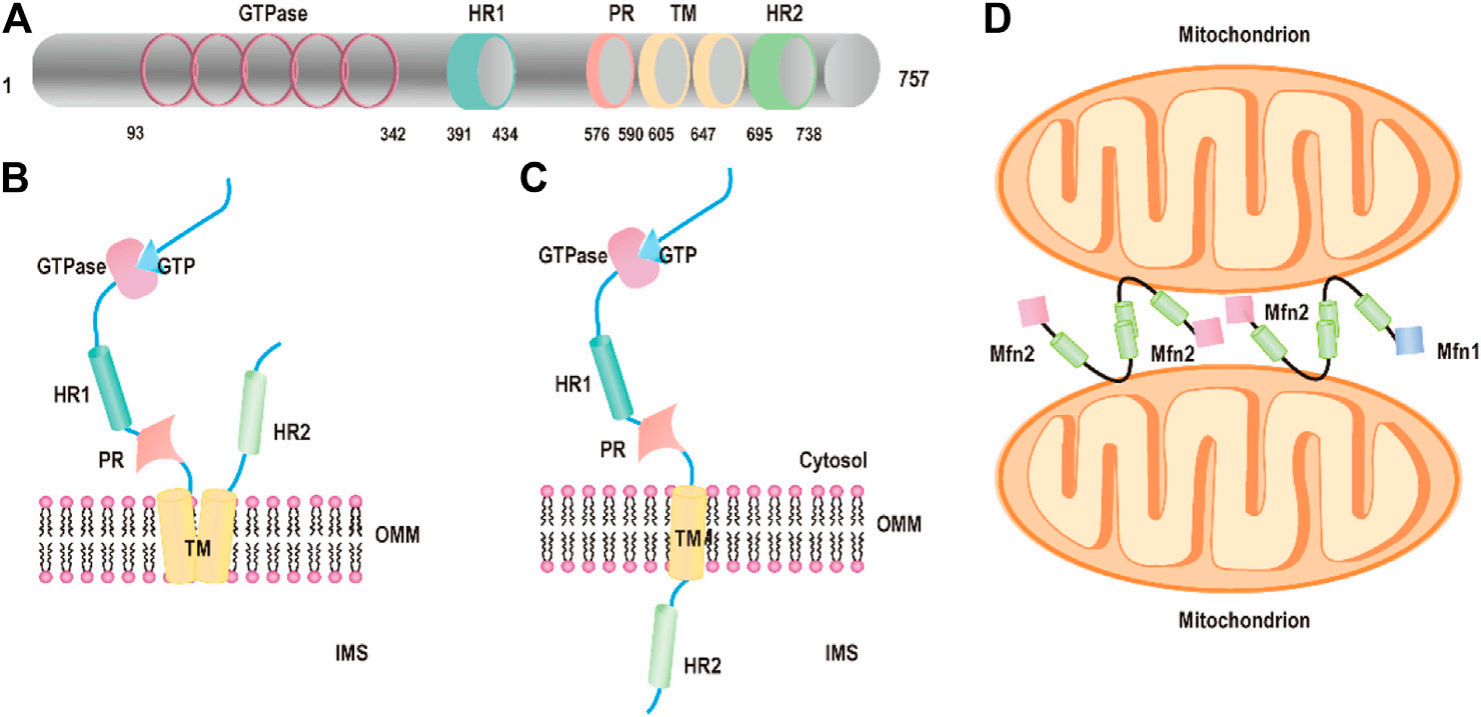
Structure of Mfn2. **(A)** The functional domains of mitofusin 2: the GTPase domains, heptad repeat (HR) coiled-coil regions 1 and 2, the PR domain, and the transmembrane (TM) domain. **(B)** Schematic diagram of Mfn2 topology: P21^RAS^ domain, a proline-rich (PR) domain, the GTPase, and coiled-coil domains (HR1 and HR2) are exposed to the cytosol and transmembrane domains crosse the OMM. **(C)** Schematic diagram of revised Mfn2 topology: P21^RAS^ domain, a GTPase, a coiled-coil (HR1), and a proline-rich (PR) domain are exposed into the cytosol, a transmembrane domain spans the OMM, and a second coiled-coil (HR) C-terminal domain protrudes into the IMS. **(D)** Mitochondrial tethering is mediated by Mfn2 or Mfn1.

The first mitochondrial fusion protein was *Drosophila* fuzzy onion (Fzo) found by Margaret Fuller and colleagues in 1997 (Hales and Fuller, 1997). Mfn2, one of the human Fzo, is dynamin-like GTPases encoded by the mitofusin 2 (MFN2) gene, which is located on the outer mitochondrial membrane. Mfn2 has 757 amino acids that consist of a GTPase domain, a first coiled-coil domain (heptad repeat HR1), two C-terminal transmembrane domains (TM), and a second coiled-coil domain (heptad repeat HR2) (Chandhok et al., 2018; Filadi et al., 2018) (Figure 1A). Mfn2 shares ∼82% similar and 66% identical with mitofusin 1 (Mfn1), and their most extensive homology exists in the same relevant functional domains (Filadi et al., 2018; Santel and Fuller, 2001). The major difference between Mfn1 and Mfn2 is that Mfn2 has a unique proline-rich domain between HR1 and the TM domains, which are tightly associated with protein–protein interactions (Filadi et al., 2018). In the canonical model, the GTPase and coiled-coil domains (HR1 and HR2) are exposed to the cytosol and transmembrane domains crosse the OMM that constitutes a hairpin curve structure mediating the mitochondrial fusion process (Figure 1B). However, Mattie et al. revised the topology of Mfn2. In the revised model, the GTPase and a coiled-coil (HR1) are still in the cytosol, a single transmembrane domain spans the OMM and a second coiled-coil (HR), and the C-terminal domain resides within intermembrane space (IMS) (Giacomello and Scorrano, 2018; Mattie et al., 2018) (Figure 1C).

Mitochondrial fusion has been studied for around two decades, and numerous excellent articles are focused on the molecules and the mechanism of mitochondrial fusion. Yet, the molecular details of the processes of fusion are still unclear. First, the HR2 domains initiate tethering between adjacent mitochondria through a dimeric antiparallel coiled-coil structure forming either homodimers (Mfn1–Mfn1 or Mfn2–Mfn2) or heterodimers (Mfn1–Mfn2) (Chen et al., 2003; Koshiba et al., 2004) (Figure 1D). The dimeric antiparallel coiled-coil is 95 Å long, constituting a large interaction interface located at opposite ends of the 95 Å coiled-coil, which links two mitochondria together (Koshiba et al., 2004). After the formation of mitochondria–mitochondria tethers, the GTPase domain further promotes fusion events accomplishment. The GTPase domain induced GTP hydrolysis to self-assembly and conformational changes, which lead to membrane remodeling and the two OMMs mixings (Sloat et al., 2019). A new study shows that Mfn2 maintains dimer’s form even after GTP hydrolysis through the GTPase domain (G) interface compared with other dynamin superfamily members including Mfn1, which ensures its higher efficient membrane tethering.(Li et al., 2019).

Recent studies provide novel insights into the mechanism of mitochondrial fusion. Qi et al. analyzed the crystal structures of human Mfn1 and observed a helix bundle (HB) containing three helices extending from the GTPase and one extending from the C-terminal tail (CT) closely attach to the GTPase domain (Qi et al., 2016). Based on the structure, the fusion model is proposed that HB1 (helix bundle1) rotates upon GTP hydrolysis, which allows HB2 (helix bundle2) to bend over and further attach to the GTPase contributing to apposing membranes merge (Qi et al., 2016). In other models, Franco et al. unveiled that in the resting or inactive state, HR2 binds with HR1 of intramolecular by an antiparallel way. In an active state, HR1–HR2 binding unfolds and extends to bind with an HR2 from a different Mfn2 molecule mediating tethering of mitochondria in trans (Franco et al., 2016). HR1 and HR2 domains exposed in the cytoplasm of OMM are necessary for all the models mentioned above to complete fusion. However, Mattie et al. have found the revised model of the C-terminal HR2 domain located in IMS. In the mammalian system, levels of oxidized glutathione disulfide (GSSG) increase, where redox mediates the formation of disulfide bridges between adjacent Mfn molecules, resulting in mitochondrial fusion (Shutt et al., 2012; Mattie et al., 2018). This result provides a new conceptual understanding of the mechanisms that activate mitochondrial fusion, but a few questions still exist. For example, what are mediators of mitochondria tethering in trans for fusion? Which domain mediates mitochondrial fusion? Besides its sensitivity to redox, does the C-terminal have other special functions? Thus, further work will aim to research on how the revised model of the Mfn2 structure drives mitochondrial fusion.

## The Function of Mitofusin 2

Mfn2 was first identified by Santel and Fuller, and its crucial function is to mediate mitochondria fusion and alter mitochondrial morphology (Santel and Fuller, 2001). After 20 years of intense research, increasing studies reveal that beyond its core function, mitochondrial fusion, Mfn2 is implicated in an expanding array of mitochondrial and cellular processes, including ER–mitochondria contacts (de Brito and Scorrano, 2008), cell apoptosis (Zhang et al., 2013), and autophagy (Chen and Dorn, 2013).

### Mitochondrial Fusion

Mitochondria are dynamic in morphology that undergo continuous remodeling by fusion and fission events. Mitochondrial fusion implicates that two mitochondria merge into an elongated one. This process permits the exchange of contents between different mitochondria and maintains genetic and biochemical uniformity *via* elimination of superoxide species and mutated DNA and the repolarization of membranes, which avoids accumulation of the mutation mitochondrial genome and loss of mitochondria (Yang et al., 2015; Bertholet et al., 2016; El-Hattab et al., 2018). Accumulating studies have reported that it has a critical role in tethering and fusing the OMM while maintaining normal mitochondria morphology. Appropriate overexpression of Mfn2 leads to mitochondrial fusion and elongation (Qin et al., 2020). However, highly overexpressed Mfn2 leads to mitochondrial aggregation around the nucleus (Huang et al., 2007). Deleting both Mfn1 and Mfn2 in mouse embryonic fibroblasts (MEFs) leads to a complete lack of mitochondrial fusion, which causes the mitochondria to become globular (Chen et al., 2003). MEFs deficient in either Mfn1 or Mfn2 genes show fragmentation of mitochondrial tubules, resulting in an aberrant mitochondrial network (Chen et al., 2003).

**FIGURE 2:**
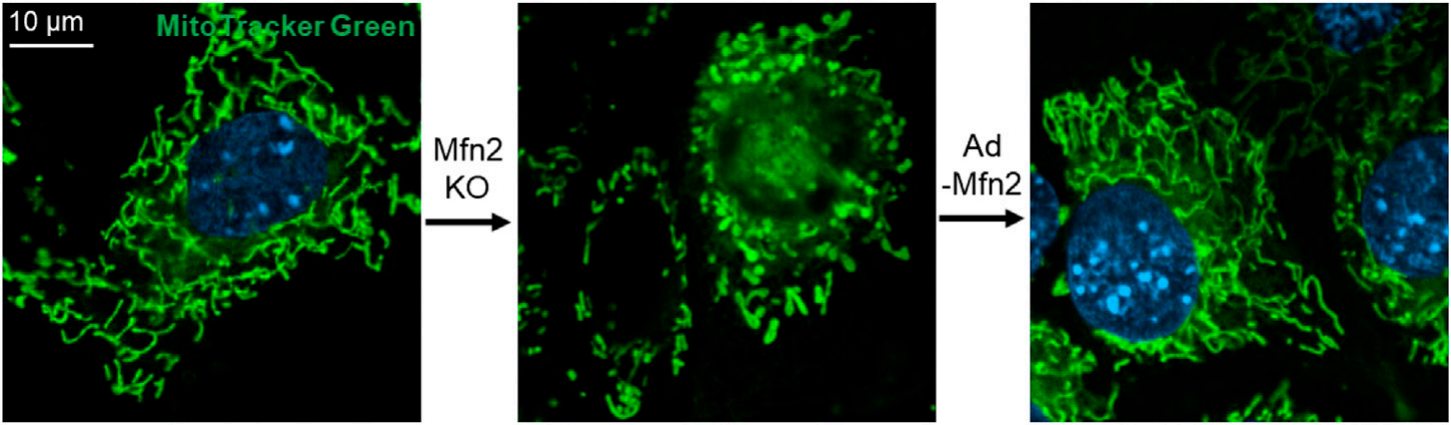
Mfn2 shapes the morphology of mitochondria in MEFs. Mfn2 deletion decreases the fusion activity that leads to mitochondrial fragmentation. The fragmented mitochondria network caused by Mfn2 deletion can be restored by adenovirus-mediated Mfn2 overexpression.

Surprisingly, the loss of either Mfn1 or Mfn2 displays different forms of fragmented mitochondria. In comparison, cells lacking Mfn1 exhibit short mitochondrial tubules or tiny mitochondrial spheres with uniform size, and cells lacking Mfn2 show some swollen spherical or short-rod mitochondria with largely different sizes and a diameter several times larger than those in wild-type (wt) cells (Figure 2) (Chen et al., 2003), which indicates a functional difference in the mitochondrial fusion. Interestingly, overexpression of Mfn1 in Mfn1 or Mfn2-deletion MEFs could restore 75–80% of cells' mitochondrial morphology (Chen et al., 2003). In contrast, overexpression of Mfn2 in Mfn2-deletion MEFs could rescue 91% cell fusion activity (Figure 2), but only 25% in Mfn1-deficient cells (Chen et al., 2003). These suggest different but partial overlapping functions between the Mfn1 and Mfn2 in the OMM fusion. Mfn1 is predominant for OMM fusion because Mfn1-harboring mitochondria show higher efficient mitochondrial membrane tethering dependent on GTPase than Mfn2-harboring mitochondria (Ishihara et al., 2004). The abundant expression of Mfn2 in the heart is non-predominant for mitochondrial fusion function, suggesting that Mfn2 integrates other biological functions.

### ER–Mitochondria Contacts

**FIGURE 3:**
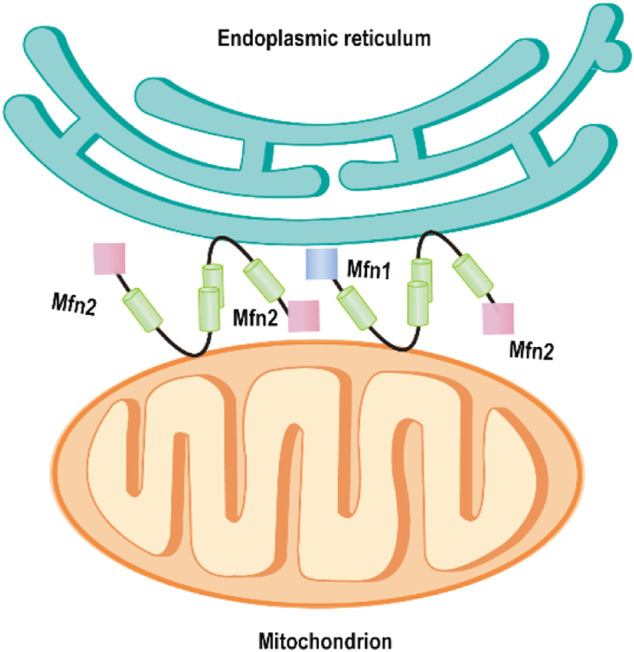
Mfn2 tethers endoplasmic reticulum to mitochondria. Mfn2 on the ER membrane interacts with Mfn2 or Mfn1 on the outer mitochondrial membrane, mediating the ER–mitochondria tethering.

The physical contacts of ER and mitochondria have been observed initially by electron microscopy (EM) in the 50 s (Bernhard et al., 1952). Yet, the character has been regarded as artifact of fixation for a long time. By 1998, Rosario and colleagues found numerous close contacts and dynamic networks between ER and mitochondria in living HeLa cells by using high resolution in three dimensions (Rizzuto et al., 1998). The mitochondria and ER are closely connected but never touch with each other. There is a gap between them, the thickness ranging from ∼10 nm up to 80–100 nm (Giacomello and Pellegrini, 2016). Therefore, mitochondria or ER-bound proteins are required for maintaining the spatial relationship between ER and mitochondria. Recently, de Brito and Scorrano firstly revealed that Mfn2 acts as a critical regulator of directly mediating mitochondrial tethering in mammals (de Brito and Scorrano, 2008). This study showed that Mfn2 proteins are enriched at resides in ER membranes, especially in contact sites between ER and mitochondria called mitochondria-associated membranes (MAMs). Mfn2 on the ER interacted with Mfn1 or 2 located in the OMM to engage in homotypic and heterotypic complexes, which mediates the two organelles tethering (de Brito and Scorrano, 2008) (Figure 3). Mfn2-deficient cells disrupted ER morphology, decreased ER–mitochondria contacts, and thus reduced mitochondrial Ca^2+^ uptake upon activation of ER Ca^2+^ release (de Brito and Scorrano, 2008). However, Pierre and his coworker observed that the percentage of the OMM engaged in the formation of close contacts (distance <20 nm) with the ER in Mfn2−/− MEFs (the same cell line used in Scorrano’s group) was more than doubled (∼4.9%) compared to its wt MEFs (∼2.2%) using the quantitative electron microscopy (EM) approach (Cosson et al., 2012). Overexpression of Mfn2 in Mfn2 KO cells significantly reduced the number of ER–mitochondria contacts. However, this study also found the reduced ER–mitochondria colocalization index (Manders’ B colocalization coefficient) in Mfn2−/− MEFs by confocal microscopy, as Scorrano’s studied. Similarly, other studies also demonstrated the increase of the structural and functional ER–mitochondria coupling in Mfn2-deficient cells or Mfn2-knockdown (KD) cells obtained by EM and confocal microscopy (Filadi et al., 2015; Leal et al., 2016) and the reduction of ER–mitochondria colocalization by fluorescence microscopy (Filadi et al., 2015). The apparent paradox is because the loss of Mfn2 alters the morphology of both the ER and mitochondria, obtained an artifact result by fluorescence microscopy. From a functional point of view, uptake of Ca^2+^ by mitochondria upon Ca^2+^ release from the ER depends on close contacts of the two organelles (Csordas et al., 2010). Therefore, the increase of their interconnection promotes this process. Riccardo et al. observed the reduced mitochondrial Ca^2+^ uptake upon ER Ca^2+^ release in Mfn2−/− MEFs. Besides, they also found that expression of the mitochondrial Ca^2+^ uniporter (MCU) was reduced by almost 50% in Mfn2^−/−^ MEFs compared with wt MEFs(Csordas et al., 2010). Therefore, it is decreased MCU not reduction of ER–mitochondria tethering that results in the reduced mitochondrial Ca^2+^ uptake. One independent experiment could support this conclusion. Acute downregulation of Mfn2 expression in wt MEFs does not affect the MCU level but induces mitochondrial Ca^2+^ rise in response to an IP3-induced ER Ca^2+^ release.

Recently, a study from Scorrano’s group further confirms the ER–mitochondria tether role for Mfn2 by employing distinct techniques as they originally reported (Naon et al., 2016). However, some conclusions are still being challenged. By using the new method: split-GFP–based contact site sensor (SPLICS), Tito’s group unraveled increased ER–mitochondria short-range (8–10 nm) contacts in acute Mfn2-KD-HeLa cells but the decreased long-range (40–50 nm) contacts (Cieri et al., 2018). Although some evidence supports the negative role of Mfn2 in the formation of close contacts (∼10 nm) between the two organelles, more evidence confirms the positive role of MFN2 in ER–mitochondria tethering. Whether Mfn2 tethers (Schneeberger et al., 2013; Li et al., 2015; Naon et al., 2016) or divides (Cosson et al., 2012; Filadi et al., 2016; Leal et al., 2016; Naon et al., 2016), ER and mitochondria remain controversial. Therefore, more useful tools, more efficient methods, and further investigations will be necessary to explore the accurate function of Mfn2 in ER and mitochondrial tethering.

### Cell Apoptosis

Mitochondria are critical for the initiation of intrinsic apoptosis. Upon apoptotic stimuli, BAX and BAK are activated and furtherly form higher-order oligomers to increase mitochondrial outer membrane permeabilization (MOMP), which is independent of the mitochondrial permeability transition pore (mPTP) opening (Bock and Tait, 2020). The increased MOMP leads to cytochrome c release into the cytosol, and subsequently, caspases are activated to initiate apoptosis. Compelling evidence exhibit that Mfn2 is integrated with proteins of the B-cell lymphoma-2 (BCL-2) family to modulate apoptosis. On apoptotic stimuli, BAX is recruited to the Mfn2-containing puncta contributing to cell apoptosis (Neuspiel et al., 2005). However, activation of BAX and release of cytochrome c are repressed in the presence of the dominant active mutants of Mfn2. Overexpression of Mfn2 promotes BAX translocation from the cytoplasm to the mitochondrial membrane, which induces apoptosis in many tumor cells, including hepatocellular carcinoma cells, breast carcinoma cells, urinary bladder carcinoma cells, and cervical carcinoma cells. These results indicate the antitumor efficacy of Mfn2 (Wu et al., 2008; Jin et al., 2011; Wang et al., 2012; Wang et al., 2018).

Overexpression of Mfn2 in hepatocellular carcinoma cells leads to calcium ion (Ca^2+^) influx from the ER. The higher mitochondrial Ca^2+^ triggers mitochondrial reactive oxygen species (ROS) generation that stimulates mPTP opening, cytochrome c release, and apoptosis (Wang et al., 2015). Mfn2 is downregulated in clinical colorectal cancer (CRC) tissues. Overexpression of Mfn2 results in a cell cycle arrest at the G2/M phase in CRC cells, and it increases the levels of activated caspase-3 and cleaved PARP (Cheng et al., 2013).

On the contrary, results from studies show the anti-apoptotic role of Mfn2. An increase of ubiquitylation and proteasomal degradation of Mfn2 in response to oxidative stress and other apoptotic stimuli leads to mitochondrial fragmentation and induced apoptotic cell death (Leboucher et al., 2012). In agreement with this result, Mfn2 overexpression could restore mitochondrial fragmentation and prevents neuronal apoptosis (Jahani-Asl et al., 2007). Similarly, Mfn2 complexed with BCL-XL maintains the anti-apoptotic function (Du et al., 2020). In brief, what is the role of Mfn2 in cell apoptosis might be context-dependent (types of cells, different apoptosis stimuli, and the interaction of distinct molecules).

### Mitophagy

**FIGURE 4:**
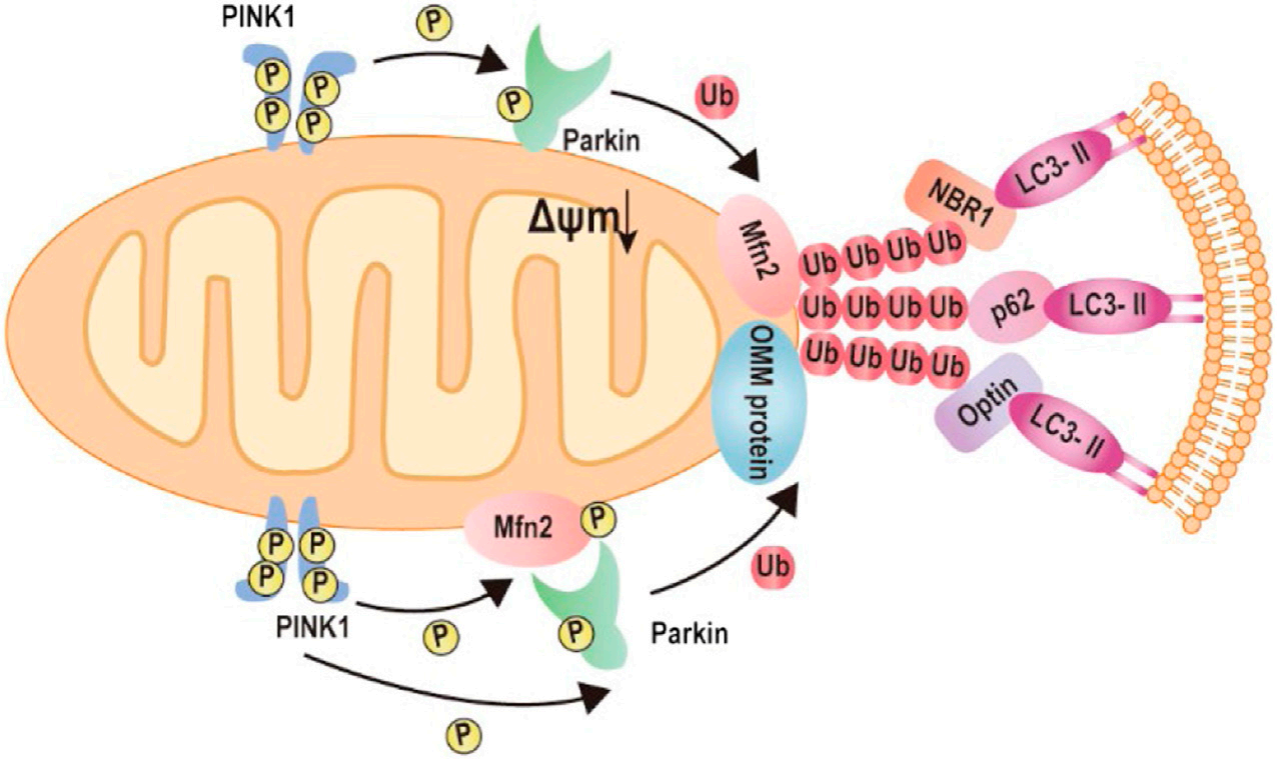
Mechanism of Mfn2-mediated mitophagy. 1) Upon mitochondrial depolarization, the kinase PINK1 accumulates at the mitochondrial outer membrane and initiates phosphorylation and recruitment of Parkin. Activated Parkin ubiquitylates Mfn2. The formation of ubiquitin chains on mitochondrial surface proteins promotes its binding to LC3-II *via* the mitochondrial receptors such as optineurin, NBR, or p62. 2) Upon mitochondrial depolarization, accumulation of kinase PINK1 phosphorylates Mfn2, which recruits Parkin to the mitochondrial outer membrane. Parkin phosphorylated by PKIN1 ubiquitylates several outer mitochondrial membrane proteins. The formation of ubiquitin chains on mitochondrial surface proteins promotes its binding to LC3-II *via* the mitochondrial receptors such as optineurin, NBR, or p62.

In order to maintain a healthy mitochondrial network and function in response to all sorts of stress, the mitochondrial quality control mechanism needs to be activated in cells, termed mitophagy (Green and Van Houten, 2011; Gong et al., 2015). Mitophagy is a cellular mechanism that selectively eliminates damaged, dysfunctional mitochondria. In this process, autophagosomes engulf mitochondria, transfer to lysosomes, and subsequently fuse with the lysosome, where degradation takes place. Studies show Mfn2 has associated with the increase in autophagosome formation and fusion of autophagosome–lysosome, suggesting that Mfn2 is a mediator of mitophagy (Sebastian et al., 2016; Peng et al., 2018). Consistently, Mfn2-KO in specific tissue such as the heart and brain directly impaired mitophagy and caused the accumulation of damaged mitochondria (Lee et al., 2012; Chen et al., 2014). The molecular mechanism of Mfn2-mediated mitophagy is uncovered by Dr. Dorn’s lab in 2013 (Chen and Dorn, 2013). The degradation of PINK1 in the depolarized mitochondria matrix will lead to PINK1 accumulates at the OMM. The phosphorylated Mfn2 at T111 and S442 by PINK1 as the Parkin receptor recruits Parkin to translocate to OMM. Subsequently, Parkin is activated *via* PINK1-mediated Ser65 phosphorylation in the Parkin ubiquitin domain. Activation of Parkin ubiquitinates MFN2 and other OMM proteins (Gegg et al., 2010; Poole et al., 2010; Tanaka et al., 2010; Ziviani et al., 2010). Furthermore, the ubiquitinated proteins bind to LC3-II *via* specific autophagy-related receptors, such as optineurin (optin), NBR1, and p62, to form autophagosomes. The formation of autophagosomes abrogates mitochondrial fusion events, which leads to mitochondrial fragmentation (Chen and Dorn, 2013; Lazarou et al., 2015; Khaminets et al., 2016; McLelland et al., 2018) (Figure 4).

## Cardiac Hypertrophy and Heart Failure

Cardiac hypertrophy (CH) occurs in a number of disease states in response to increased cardiac workload and can readily progress to ventricular dilatation, contractile dysfunction, and heart failure. Although there is limited literature that decodes the function of Mfn2 in heart failure, it has been reported that Mfn2 has associated with the onset and progression of cardiac hypertrophy and heart failure. Mfn2 expression is decreased in various rat models of cardiac hypertrophy, including MI and TAC (Fang et al., 2007). Similarly, Mfn2 expression is downregulated in Ang II-induced or PE-induced cardiomyocyte hypertrophy. Upregulation of Mfn2 could inhibit the hypertrophic progression (Yu et al., 2011; Guan et al., 2016; Sun et al., 2019).

Mfn2 deletion interrupts multiple molecular signal pathways that gradually lead to cardiac vulnerability and dysfunction. Recently, genetic ablation mice are used to explore the function of Mfn2 in cardiac hypertrophy and heart failure. A report from Dorn’s group exhibits that hearts deficient in Mfn1 and Mfn2 contribute to progressive dilated cardiomyopathy at five weeks and heart failure from seven to eight weeks (Chen et al., 2011). Furthermore, dilated cardiomyopathy is observed in mice lacking Mfn2 at 16 weeks, and the severity increases with age (Chen and Dorn, 2013). However, another study shows that cardiac deletion of Mfn1 and Mfn2 in mice is no sign of cardiac hypertrophy at 8–10 weeks, with normal left ventricular volume and normal contractile performance (Hall et al., 2016). Conditional deletion of Mfn2 only leads to mild myocyte hypertrophy accompanied by mild deterioration of left ventricular function and does not develop heart failure (Papanicolaou et al., 2011). Mfn2 deletion or Mfn1 and Mfn2 deletion protect the heart against ischemia and reperfusion injury (Papanicolaou et al., 2011; Hall et al., 2016). Genetic deletion mice from different study groups display distinct phenotypes, which might attribute to deletion methods, the location and timing of Mfn2 deletion, the genetic background, and the ages of the mice.

**FIGURE 5:**
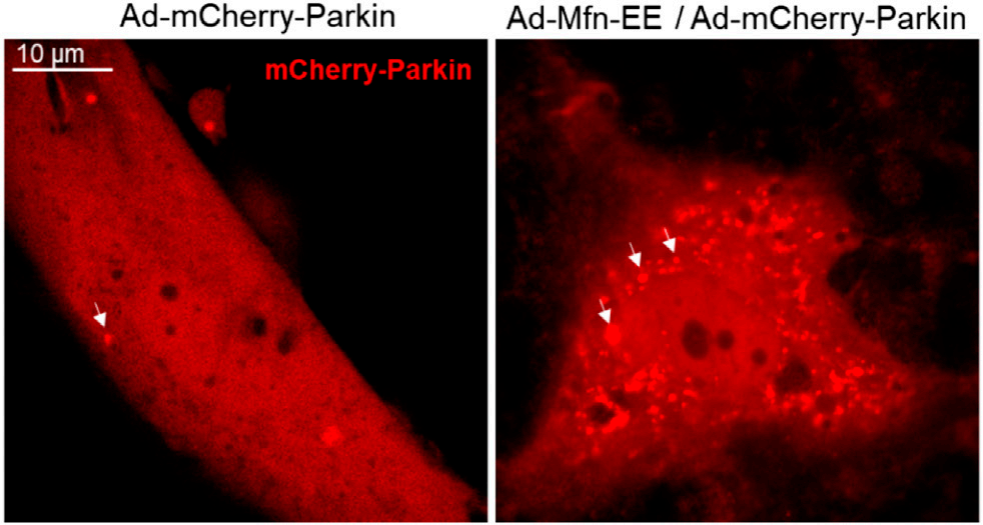
Phosphorylated Mfn2 recruits Parkin to OMM. Mfn2 Thr^111^ (T111) and Ser^442^ (S442) were replaced by Glu (E) that mimics PINK1 phosphorylation of Mfn2 (T111E/S442E) conferred it PINK1-independent binding activity to Parkin.

Mfn2 plays a critical mediator in the development of cardiac hypertrophy and heart failure *via* regulation of various biological processes, including mitochondrial fusion, mitophagy, and apoptosis. Vivo studies demonstrated that specific deletion of Mfn2 in cardiomyocytes impaired mitochondrial fusion and modest cardiac hypertrophy (Papanicolaou et al., 2011). Similarly, *in vitro* studies observed that Mfn2 was decreased in Ang II-induced cardiac myocytes hypertrophy, accompanied by the change of mitochondria morphology from the filament to short and small. The aspect ratio of the mitochondria is also reduced (Xiong et al., 2019; Yu et al., 2011). Mfn2 overexpression on Ang II-induced cardiomyocyte rescue mitochondrial fusion, which resulted in two short and small mitochondria to form large mitochondria and attenuated myocyte hypertrophy (Xiong et al., 2019). Cardiac deletion of Mfn2 in mice increases the proportion of enlarged mitochondria and impairs mitophagy that leads to cardiomyopathy. Upon mitochondria depolarization, PINK1-phosphorylated Mfn2 recruits Parkin to the OMM and facilitates mitophagy (Figure 5). Mfn2 ablation defects Parkin signaling, initiating aberrant mitophagy (Chen and Dorn, 2013). Interestingly, the effects of Mfn2 overexpression ameliorated Ang II-induced cardiac hypertrophy *via* facilitating Parkin translocation and phosphorylation, triggering mitophagy (Xiong et al., 2019). Besides, autophagosome–lysosome fusion is impaired in Mfn2-deficient mice, leading to accumulation of autophagosomes and progressive heart failure (Zhao et al., 2012). Mfn2 mediates autophagosome–lysosome fusion in the heart *via* attracting and binding RAB7 to the autophagosomal membrane (Zhao et al., 2012).

Besides, Mfn2 expression is associated with proliferation and apoptosis of VSMCs and myocytes in cardiac hypertrophy (Chen et al., 2004). Neonatal rat cardiomyocytes treated with H_2_O_2_ inducing oxidative stress lead to concurrent increases in Mfn2 expression and apoptosis (Guo et al., 2007; Shen et al., 2007). Further study shows that upregulation of Mfn2 significantly increases caspase-9, caspase-3, and BAX/BCL-2 ratio, initiates cytochrome c release, and decreases the level of phosphorylated Akt, thereby triggering apoptosis (Guo et al., 2007). Additionally, Papanicolaou et al. observed that cardiomyocytes by genetically deleting Mfn2 can be resistant to cell death, owing to more tolerance to Ca^2+^-induced MPTP opening (Papanicolaou et al., 2011). Mfn2 could regulate ER–mitochondria tethering, which is related to Ca^2+^ influx. ER and mitochondria contacts are more close, and Ca^2+^ influx in the mitochondrial matrix is highly increased. High-level mitochondrial Ca^2+^ facilitates the opening of MPTP which leads to apoptosis (Filadi et al., 2015). Cumulatively, these results indicate that Mfn2 is a necessary component for cardiac hypertrophy and heart failure.

Although the functions of Mfn2 in cardiac hypertrophy and heart failure are far more complex than what we have yet known, enhancing Mfn2 function or upregulating expression may be useful as a therapeutic strategy for cardiac hypertrophy and heart failure. Recently, reporters have observed that 6-phenylhexanamide derivative Mfn2 activators or Mfn2 agonists could enhance Mfn2 function for the treatment of diseases (Rocha et al., 2018; Dang et al., 2020). Besides, sex hormones (estrogen and testosterone) also upregulate Mfn1 and Mfn2 *via* the regulation of PGC1α in cardiac tissue (Lynch et al., 2020). Although, direct modulation of Mfn1 and Mfn2 by the sex hormones is still unclear in heart failure, several studies have indicated estrogen and testosterone are cardioprotective factors (Volterrani et al., 2012; Lynch et al., 2020). It is also convenient for the patient to monitor and analyze the concentration of hormones by modern medical techniques.

## Concluding Remarks and Perspectives

The role of Mfn2 is essential for heart diseases, owing to its ability to regulate mitochondria fusion, ER–mitochondria contacts, cell metabolism, apoptosis, and autophagy. Alteration of Mfn2 expression or dysfunctions of Mfn2 has been observed in heart diseases. Some studies indicate that overexpression of Mfn2 in heart diseases, including heart failure and myocardial ischemia, could attenuate cardiac hypertrophy and dysfunction in response to various stresses. Other studies show that deletion of Mfn2 in cardiac myocytes is able to protect against ischemia and reperfusion injury. Therefore, further studies are needed to explore detailed molecular mechanisms of Mfn2 in cardiovascular diseases, which might provide a novel therapeutic target for patients.

## Glossary

Ang II: angiotensin II
BCL-2: B-cell lymphoma-2
CDK: cyclin-dependent kinases
CT: C-terminal tail
Drp1: dynamin-related protein 1
EM: electron microscopy
ER: endoplasmic reticulum
Fzo: Drosophila fuzzy onion
HB: helix bundle
GSSG: glutathione disulfide
KD: knockdown
MAM: mitochondria-associated membranes
MCU: mitochondrial Ca2+ uniporter
MEFs: embryonic fibroblasts
MFN1: mitofusin 1
MFN2: mitofusin 2
MFNs: mitofusins
MI: myocardial infarction
MOMP: mitochondrial outer membrane permeabilization
OMM: outer mitochondrial membrane
Opa1: optic atrophy 1
Optin: optineurin
PE: phenylephedrine
SHR: spontaneously hypertensive rats
SPLICS: split-GFP–based contact site sensor
TAC: transverse aortic constriction
TM: transmembrane domains
VSMCs: vascular smooth muscle cells
WKY: Wistar Kyoto rat
WT: wild type
